# Values and preferences influencing willingness to change red and processed meat consumption in response to evidence-based information: a mixed methods study

**DOI:** 10.1017/S1368980022000866

**Published:** 2022-08

**Authors:** Anna Prokop-Dorner, Aleksandra Piłat-Kobla, Joanna Zając, Michalina Luśtyk, Claudia Valli, Aneta Łapczuk, Monika Brzyska, Bradley Johnston, Dena Zera, Gordon Guyatt, Pablo Alonso-Coello, Malgorzata M Bala

**Affiliations:** 1 Department of Medical Sociology, Chair of Epidemiology and Preventive Medicine, Jagiellonian University Medical College, 31-034 Krakow, Poland; 2 Department of Hygiene and Dietetics, Jagiellonian University Medical College, Krakow, Poland; 3 Department of Paediatrics, Obstetrics, Gynaecology and Preventive Medicine, Universidad Autónoma de Barcelona, Barcelona, Spain; 4 Iberoamerican Cochrane Centre, Biomedical Research Institute San Pau (IIB Sant Pau), Barcelona, Spain; 5 College of Agriculture and Life Scinces, Texas A&M University, College Station, TX, USA; 6 Department of Epidemiology & Biostatistics, School of Public Health, Texas A&M University, College Station, TX, USA; 7 Department of Biomedical Informatics, Harvard Medical School, Boston, MA, USA; 8 Department of Health Research Methods, Evidence, and Impact, McMaster University, Hamilton, ON, Canada; 9 Department of Medicine, McMaster University, Hamilton, ON, Canada; 10 CIBER de Epidemiología y Salud Pública (CIBERESP), Barcelona, Spain

**Keywords:** Values and preferences, Meat consumption, Evidence-based health information, Cancer health risk, Mixed methods

## Abstract

**Objectives:**

The aim of the study is (1) to assess the extent to which omnivores are willing to stop or reduce their consumption of red and processed meat in response to evidence-based information regarding the possible reduction of cancer mortality and incidence achieved by dietary modification; (2) to identify socio-demographic categories associated with higher willingness to change meat consumption and (3) to understand the motives facilitating and hindering such a change.

**Design:**

During an initial computer-assisted web interview, respondents were presented with scenarios containing the estimates of the absolute risk reduction in overall cancer incidence and mortality tailored to their declared level of red and processed meat consumption. Respondents were asked whether they would stop or reduce their average meat consumption based on the information provided. Their dietary choices were assessed at 6-month follow-up. Additionally, we conducted semi-structured interviews to better understand the rationale for dietary practices and the perception of health information.

**Participants:**

The study was conducted among students and staff of three universities in Krakow, Poland.

**Results:**

Most of the 513 respondents were unwilling to change their consumption habits. We found gender to be a significant predictor of the willingness. Finally, we identified four themes reflecting key motives that determined meat consumption preferences: the importance of taste and texture, health consciousness, the habitual nature of cooking and persistence of omnivorous habits.

**Conclusions:**

When faced with health information about the uncertain reduction in the risk of cancer mortality and incidence, the vast majority of study participants were unwilling to introduce changes in their consumption habits.

Over the past decades, medical and media discourse have popularised various nutrients, foods and dietary patterns. Different social factors, such as national^(1)^ and international health agencies^(2)^, medical associations^(3)^ and media influencers^(4–6)^, promote particular nutrients, food products or dietary habits as either beneficial or harmful to one’s health. This includes the consumption of red and processed meat, which is the focus of this paper. Recently, critics have questioned the evidence informing recommendations and the extent to which they reflect the values and preferences of the population targets^(7,8)^. Firstly, political pressures from food companies influence both nutritional studies^(9)^ and dietary guidelines and governmental policies regarding nutrition^(10)^. Secondly, while there is a consensus that dietary guidelines—like any other guidelines—should reflect the highest-quality available evidence^(7,11)^, evidence regarding the impact of diet on long-term health outcomes is typically of low or very low quality. This is certainly true for data on the association of red meat consumption with cancer and cardiovascular risk^(12–15)^.

At the other end of dietary recommendations, guidelines have typically neglected the impact of well-established eating habits. They have also taken for granted changes in consumption that well-informed individuals would be willing to make in response to possible health benefits^(16)^. While the significance of values and preferences is often minimised when developing dietary recommendations, they are crucial when the evidence is uncertain and when effect estimates are small. The practice of evidence-based nutrition should involve not only awareness of the best available evidence and guidance to certainty in estimates of test properties, patients’ prognosis or treatment effects but also acknowledging individual and societal values and preferences^(17–19)^. We define them as *the collection of goals, expectations, predispositions, beliefs, and abilities and resources to make the changes that individuals have for certain decisions that may influence their outcomes* [17:196]. In social sciences, values are conceptualised as a key component of culture and defined as abstract concepts acquired through the process of primary and secondary socialisation, which guides individuals’ beliefs and behaviours^(20)^. Taken for granted conceptions about food, taste and health, as well as practices of satisfying hunger, celebrating festive occasions or marking social identity define lifestyle and impact health behaviours and may therefore impede introducing changes thereinto^(10)^.

The authors of a systematic review on values and preferences concluded that omnivores are attached to their meat consumption habits and are unwilling to change even when faced with potentially undesirable health effects; however, the available evidence was of low quality^(21)^. Nevertheless, given that this constituted the highest quality of evidence available, it played a key role in developing a weak recommendation for continuing the current levels of red and processed meat consumption^(22)^, a recommendation that proved controversial. The primary reason for the low-quality evidence is that no study had directly addressed the question at hand: When informed of the possible health benefits of reducing meat consumption, to what extent would individuals currently regularly eating meat be willing to modify their diet? Therefore, we undertook this mixed methods study to directly address the above question and explore peoples’ motivation for their dietary choices.

## The socio-cultural context and dietary patterns in Poland

Despite disadvantageous health indicators (45·7 % of women and 62·2 % of men in Poland are overweight, as compared with the average for the European Union countries of 42·7 % and 57·1 %, respectively)^(23)^, most Poles (80 %) are convinced about their dietary habits being healthy or very healthy and only fewer than one-fifth describes own diet as unhealthy (17 %)^(24)^. Poles are among the six least physically active nations in the European Union^(24)^. Polish dietary patterns studied in the last two decades revealed changes with regard to increased meat intake^(25)^ and reproducing traditional culinary practices associated with Eastern Europe^(26)^. A comparison of dietary patterns in three countries carried out in the HAPIEE study revealed that a high adherence to the traditional Eastern European diet, consisting of (1) bread and grain products, (2) potato, (3) legumes, (4) storable vegetables, (5) preserved fruits and vegetables, (6) dairy products and egg, (7) poultry, (8) processed meat products and (9) lard for cooking, was considerably higher in the Polish sample compared with the Russian and Czech samples^(26)^. A Polish daily menu includes bread at least once a day (90 % of Poles) and fresh and frozen vegetables and fruits (62 % and 61 %, respectively), meat and meat products (36 %) and fish (1 %)^(24)^. In 2018, the consumption of meat per capita in Poland (62·4 kg in total)^(27)^ was somewhat higher than in other European countries, such as Germany (61·3 kg), Italy (58·6 kg), Spain (59 kg) and the United Kingdom (54·5 kg)^(28)^. Reduction in the consumption of some products has been declared by three in ten adult Poles, of whom 11 % excluded meat. The most crucial reasons for Poles for being on diet were willingness to hone nutrition habits (35 %), illness (34 %), weight loss (32 %) or doctor’s recommendations (23 %). Those motivated by medical doctors mentioned having CVD (28 %), diabetes (25 %) and alimentary tract disorders (21 %). Women more often than men followed some diets (31 % and 26 %, respectively), and female consumers were typically more consistent with their diet (18 % *v*. 15 %). Dieting was more common among individuals with higher education (38 %) and younger respondents aged 18–34 years (36–38 %)^(24)^. The research question of this study was addressed in the context of the Polish culinary culture.

## Methods

**Fig. 1:**
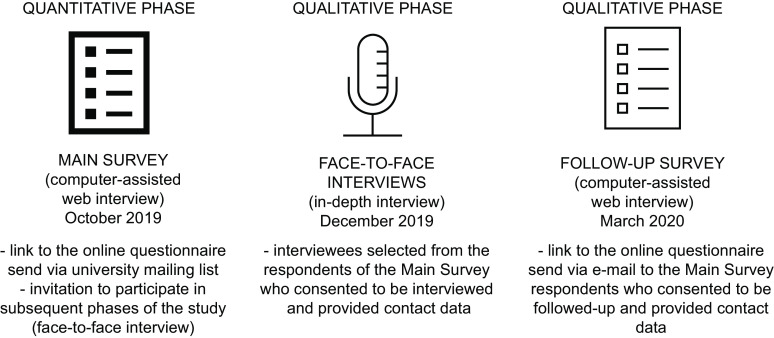
Study phases and general information on the recruitment of participants

The aims of this cross-sectional mixed methods study^(29)^ were as follows: (1) to assess the extent to which omnivores are willing to reduce their consumption of red and processed meat in response to evidence-based information regarding the reduction of cancer mortality and incidence they might achieve by dietary modification; (2) to identify which socio-demographic categories are associated with higher willingness to change their meat consumption and (3) to understand the motives facilitating and hindering such a dietary change. We combined quantitative data collected during an online survey (computer-assisted web interview) among students, staff and faculty members of three universities in Krakow, Poland, with qualitative data from in-depth interviews carried out among a purposively selected sample of survey respondents (Fig. 1). The choice of the population was based on the assumption that individuals with a higher educational status demonstrate the highest levels of health literacy^(30)^ and more often declare healthy dietary habits^(24)^. To diversify the sample, the study involved three universities, including both general and technical universities. The quantitative measurement, conducted twice at an interval of 6 months, enabled us to describe the dominant preferences regarding meat consumption, while the qualitative study facilitated the understanding of the reasons and beliefs underlying willingness or unwillingness to change diet.

The study protocol^(31)^ was developed as part of an international cooperation within the framework of NutriRECS^(22)^. The study was piloted with thirty-two individuals from two Canadian provinces^(32)^. All study participants provided informed consent for inclusion in the study.

### Quantitative phase

#### Recruitment of respondents

According to the domestic deliveries and consumption report in Poland, the average intake of both unprocessed and processed meat in 2017 was 115 g/d (9 portions/week)^(33)^. Based on these data, we defined the lower threshold for the average intake of both red and processed meat as three portions/week and included only those respondents who consumed at least three portions of either type of meat. Furthermore, prior to completing the questionnaire, we verified respondents’ eligibility for the study. The online questionnaire was completed only by individuals who did not declare any of the following: active cancer, severe CVD or pregnancy, unwillingness or inability to provide informed consent^(31)^. At the stage of the analysis, we excluded professionally inactive individuals (eight respondents).

The invitation to participate in the survey, together with the background study and contact information as well as a link to the questionnaire, was sent to scientific and administrative employees and students via the electronic mailing systems of the three universities.

At the end of the main survey’s questionnaire, we asked respondents to participate in subsequent parts of the study, including an in-depth interview and/or a brief follow-up survey. Individuals who positively responded to the invitation were contacted via phone or email. Participants were not offered any incentives.

#### Research instrument

The questionnaires employed in the main and follow-up phases were developed by an interdisciplinary research team previously involved in the systematic review on values and preferences related to meat consumption^(21)^. The questionnaire used in the main survey explained the types of meat addressed in the survey. It was divided into three parts:

1. Socio-demographic: it consisted of twenty-two items addressing respondents’ sex, age, education level, marital status, religion, ethnicity, weight, height, physical activity, chronic illnesses and a family history of cancer.

2. Meat consumption beliefs and behaviour: it consisted of twenty-three questions regarding red and processed meat consumption. Respondents specified how many portions of each type of meat they ate in a week and which products from each type they ate the most. The questionnaire provided a detailed description of the portions of unprocessed red meat^(31)^. It also contained pictures illustrating both types of meat and portion sizes (see Supplementary Material 1). Next, respondents selected three main reasons determining their decision about meat consumption out of eleven categories, including price, health issues, taste, availability, family preferences, tradition, religion, preparation time, social context, animal welfare, environmental issues and others. The subsequent six items asked about food buying practices and another four items referred to satisfaction with the current diet.

3. Direct choice exercise: it assessed willingness to reduce or eliminate red and processed meat consumption when faced with information about the possible health effects related to the change in meat consumption habits. This section included a description of each outcome (cancer mortality and incidence, see Supplementary Material 3 Part Three) as well as absolute risk reductions for overall cancer mortality and incidence if respondents eliminated or reduced their meat consumption. The latter was based on absolute reductions visually displayed per 1000 people using the MagicApp software derived from a systematic review with dose-response meta-analysis^(15,34)^ (see Supplementary Material 2). It also included a video and a written explanation of how respondents should interpret the data as well as a 7-point scale assessing respondents’ willingness to change meat consumption (see Supplementary 3 Part Three). We also presented the corresponding certainty of evidence for potential health benefits. When the respondent expressed unwillingness to eliminate meat consumption after being presented with evidence for potential absolute risk reduction in adverse health outcomes, we asked about their willingness to reduce consumption. We provided respondents with the scenario of reduction in health risk tailored to their level of consumption declared in questionnaire Part One.

The questionnaire used during the follow-up stage was aimed to establish whether respondents had made any changes in meat consumption since participation in the main survey^(31)^. Those who had introduced any changes to their meat intake pattern were presented with four further questions regarding the reasons for such a change, including health outcome information provided previously.

The original English language version of the questionnaire and additional materials were translated into Polish and piloted on a sample of fifteen employees. The self-administered questionnaires were distributed electronically via university mailing lists (main survey) or directly to respondents’ email addresses (follow-up survey). Both quantitative phases were carried out using the LimeSurvey application dedicated to online surveys^(35)^.

### Quantitative data analysis

Frequencies of participants’ characteristics were described. The distribution of the continuous variables ‘willingness to reduce meat consumption in the face of undesirable cancer health risks’ and ‘willingness to eliminate meat consumption in the face of undesirable cancer health risks’ was described using median and IQR. Histograms were used to plot the percentage frequency of score occurrences. Moreover, we conducted an exploratory logistic regression analysis using the above variables as categorical variables: willingness (≥ 5 points on the 7-point scale) and unwillingness (≤ 4 points on the 7-point scale) to change meat consumption and calculated the OR and the associated 95 % CI for willingness to eliminate or reduce meat consumption. The frequency and proportion of participants from the follow-up sample who introduced any changes in their meat consumption habits at 6 months was calculated. The data were analysed using IBM SPSS Statistics (Version 26) predictive analytics software^(36)^.

### Qualitative phase

#### Research instrument

The semi-structured interviews were carried out by two qualitative researchers according to an interview guide (see Supplementary material 4) developed within the international cooperation^(31)^. The interview guide covered questions on the key motives determining red and processed meat consumption and the evaluation of the health information presented in the survey. The interview guide was translated into Polish and piloted on a small sample.

During the face-to-face interviews, interviewees were reminded their responses regarding their reasons for meat consumption given in the questionnaire and encouraged to elaborate on them. The cultural context of shaping individuals’ beliefs and their related dietary practices typically results in people taking them for granted. For instance, many of the individuals’ culinary and consumption practices are habitual. Such culturally acquired values and preferences linked to dietary habits are challenging to study. Therefore, asking indirect questions and probing participants about what may be taken for granted is the most optimal qualitative research strategy to learn about people’s values and preferences. Talking about their dietary habits and asking participants about the reasons why they consume as well as maintain or change them provided insight into the cultural norms underlying those values and preferences. The interviewers were probing the study participants to provide examples of their particular dietary practices and social situations involving meat consumption. Interviewees were asked about their impression on the information on possible health benefits related to the change in meat consumption presented in the main survey. Finally, they were encouraged to establish what kind of data could encourage any potential change in their meat consumption.

#### Sampling

From among those who agreed to participate in the subsequent phases of the study, we purposefully selected individuals based on their socio-demographic characteristics (i.e. sex) and declared meat consumption. We made efforts to gather a heterogeneous sample of women and men with low (a less than average number of portions of red and processed meat) and high meat consumption (an average number of portions of red and processed meat or more). Of the individuals with whom the interviews were arranged, two cancelled the appointments without providing a reason. The recruitment process was discontinued when data saturation was reached (i.e. researchers provided repeated evidence for their conceptual categories, and further data collection would not generate any new conceptual insights)^(37)^.

#### Qualitative data analysis

The interviews were audio-recorded and transcribed verbatim. The analysis was conducted in an ongoing manner. We applied the analytical approach of thematic analysis by Boyatzis^(38)^. Both researchers kept their observations in a logbook throughout the process of data collection and analysis. The purpose of the qualitative analysis is to emphasise how data fit together as a whole and bring together the context and meaning^(39)^. It requires starting with data immersion and leads to familiarity with the content of the transcripts and enables noticing common themes. Next, we coded the gathered material using a mixed approach. We developed the codebook partly deductively and partly inductively, when reading through the data and identifying emergent themes expanding the preformulated list. In the next step, we focused on working through code reports aggregating segments of transcripts coded with the same code. We displayed data to capture the variations of themes. We also applied some visual tools to overview how many segments from each interview were assigned specific codes and to distinguish nuances of the themes. It enabled us to describe the individual values and preferences of the interviewees and to identify broader patterns. We applied the constant comparison method to understand relations between themes. Throughout the analysis, we wrote summaries and memos to develop a deeper understanding of the patterns, form hypothesis and interpretations, as well as document our insights. The MAXQDA2018 software was used in all analytical procedures^(40)^.

## Results

### Quantitative phase

#### Response rate and socio-demographic characteristics of participants

The link to the survey was accessed by 3932 individuals, and 2097 (53 %) respondents filled out the questionnaire. We included 513 respondents in the main survey (mean age, 32·91 ± 12·63 years), and 333 respondents consented to participate in the follow-up. Of these, 176 (52·85 %) responded to the follow-up survey (Table 1).

**Table 1:** Socio-demographic characteristics of the respondents participating in the main survey in October 2019 and in a follow-up study in March 2020

	Main survey		Follow-up survey	
	*n*	%	*n*	%
Sex				
Male	204	39·8	65	36·9
Female	309	60·2	111	63·1
Age				
0–25	202	39·5	62	35·2
26–40	174	34·0	68	38·6
≥41	136	26·6	46	26·1
Education				
Elementary/secondary	62	12·1	17	9·7
Post-secondary	73	14·2	20	11·4
Higher	378	73·7	139	79·0
Occupational status				
Employee	318	62·0	113	64·2
Student	195	38·0	63	35·8
Religion				
Christianity	381	74·3	127	72·2
Other	9	1·8	5	2·8
None	123	24·0	44	25·0
Family history of cancer				
Yes	253	49·3		
No/I don’t know	260	50·7		

#### Patterns of unprocessed and processed meat consumption

As shown in Table 2, processed meat was more often consumed than red meat. Meat consumption was significantly lower in women compared with men (*P* < 0·001). Every tenth woman (11·6 %) and every third man (30·4 %) declared eating more than four portions of red meat per week, while 29·1 % (3/10) of women and 44·1 % (4/10) of men declared consuming more than four portions of processed meat per week.

**Table 2:** Self-reported consumption of red and processed meat per week (*P* < 0·001), *n* 513

Meat consumption per week	Unprocessed red meat*				Processed meat**			
	Men *n* 204		Women *n* 309		Men *n* 205		Women *n* 311	
	*n*	%	*n*	%	*n*	%	*n*	%
< 3 portions	75	36·8	185	59·9	52	25·5	121	39·2
3–4 portions	67	32·8	88	28·5	62	30·4	98	31·7
5–8 portions	51	25·0	35	11·3	59	28·9	72	23·3
9–12 portions	9	4·4	1	0·3	16	7·8	10	3·2
> 13 portions	2	1·0	0	0·0	15	7·4	8	2·6

Data are presented as number (percentage).

* *P* < 0·001 (Fisher’s exact test).

** *P* < 0·001.

The main factors determining the decision about red and processed meat consumption were similar: taste (76·2 % and 74·5 % for red and processed meat, respectively), family preferences (35·5 % and 32·6 %, respectively), price (28·1 % and 34·3 %, respectively), availability (25·9 % and 38·2 %, respectively), health (23·2 % and 13·3 %, respectively) and preparation time (20·1 % and 35·5 % respectively). The taste of red meat was significantly more important for men than for women, while the preparation time was more important for women than for men. For both red and processed meat, the preferences of family members were more important for women than for men (*P* < 0·001 and *P* = 0·01, respectively), and for red meat, they were more important for older respondents than for younger ones (*P* = 0·001). Easy availability and short preparation time of processed meat were more important for the youngest respondents than for the other age groups (Table 3).

**Table 3:** Key reasons for meat consumption by socio-demographic category

Reasons for red meat consumption	Socio-demographic categories																
	Sex, *n* (%)					Occupation, *n* (%)					Age group, *n* (%)						
	Male		Female		*P* value	Employee		Student		*P* value	0–25		26–40		≥41		*P* value
Price	53	26·0	91	29·4	0·45	69	21·7	75	38·5	<0·001*	77	38·1	42	24·1	24	17·6	<0·001*
Health issues	48	23·5	71	23·0	0·97	83	26·1	36	18·5	0·06	39	19·3	43	24·7	37	27·2	0·21
Taste	170	83·3	221	71·5	0·003*	233	73·3	158	81·0	0·06	163	80·7	131	75·3	96	70·6	0·10
Availability	55	27·0	78	25·2	0·74	74	23·3	59	30·3	0·10	60	29·7	45	25·9	28	20·6	0·17
Family preferences	52	25·5	130	42·1	<0·001*	120	37·7	62	31·8	0·20	64	31·7	60	34·5	58	42·6	0·11
Tradition	29	14·2	39	12·6	0·70	45	14·2	23	11·8	0·53	23	11·4	25	14·4	20	14·7	0·59
Religion	2	1·0	0	0·0	0·16†	1	0·3	1	0·5	0·99†	1	0·5	0	0·0	1	0·7	0·73†
Preparation time	30	14·7	73	23·6	0·02*	64	20·1	39	20·0	0·99	41	20·3	30	17·2	32	23·5	0·39
Social context	30	14·7	40	12·9	0·66	46	14·5	24	12·3	0·58	25	12·4	27	15·5	17	12·5	0·62
Animal welfare	9	4·4	16	5·2	0·85	13	4·1	12	6·2	0·40	12	5·9	8	4·6	5	3·7	0·62
Environmental issues	6	2·9	14	4·5	0·50	15	4·7	5	2·6	0·32	6	3·0	8	4·6	6	4·4	0·68
Other	3	1·5	6	1·9	0·99†	4	1·3	5	2·6	0·31†	5	2·5	2	1·1	2	1·5	0·70

Reasons for processed meat consumption	Sociodemographic categories																
	Sex, *n* (%)					Occupation, *n* (%)					Age group, *n* (%)						
	Male		Female		*P* value	Employee		Student		*P* value	0–25		26–40		≥41		*P* value
Price	64	31·4	112	36·2	0·30	72	22·6	104	53·3	<0·001*	110	54·5	42	24·1	23	16·9	<0·001*
Health issues	23	11·3	45	14·6	0·35	52	16·4	16	8·2	0·01*	19	9·4	28	16·1	21	15·4	0·11
Taste	152	74·5	230	74·4	0·99	241	75·8	141	72·3	0·44	143	70·8	135	77·6	103	75·7	0·30
Availability	73	35·8	123	39·8	0·41	103	32·4	93	47·7	0·001*	102	50·5	56	32·2	38	27·9	<0·001*
Family preferences	53	26·0	114	36·9	0·01*	119	37·4	48	24·6	0·004*	47	23·3	62	35·6	58	42·6	0·001*
Tradition	10	4·9	10	3·2	0·47	15	4·7	5	2·6	0·32	5	2·5	8	4·6	7	5·1	0·39
Religion	1	0·5	0	0·0	0·40†	1	0·3	0	0·0	0·99†	0	0·0	0	0·0	1	0·7	0·27†
Preparation time	82	40·2	100	32·4	0·09	98	30·8	84	43·1	0·006*	86	42·6	61	35·1	35	25·7	0·006*
Social context	11	5·4	26	8·4	0·26	29	9·1	8	4·1	0·05	7	3·5	17	9·8	12	8·8	0·04*
Animal welfare	6	2·9	12	3·9	0·75	12	3·8	6	3·1	0·87	6	3·0	8	4·6	4	2·9	0·69†
Environmental issues	4	2·0	7	2·3	0·99†	6	1·9	5	2·6	0·76†	5	2·5	1	0·6	5	3·7	0·16†
Other	2	1·0	7	2·3	0·33	7	2·2	2	1·0	0·49†	2	1·0	5	2·9	2	1·5	0·40†

* Significant differences.

† *P* value for the Fisher’s exact test of independence.

#### Willingness to eliminate or reduce meat consumption

**Fig. 2:**
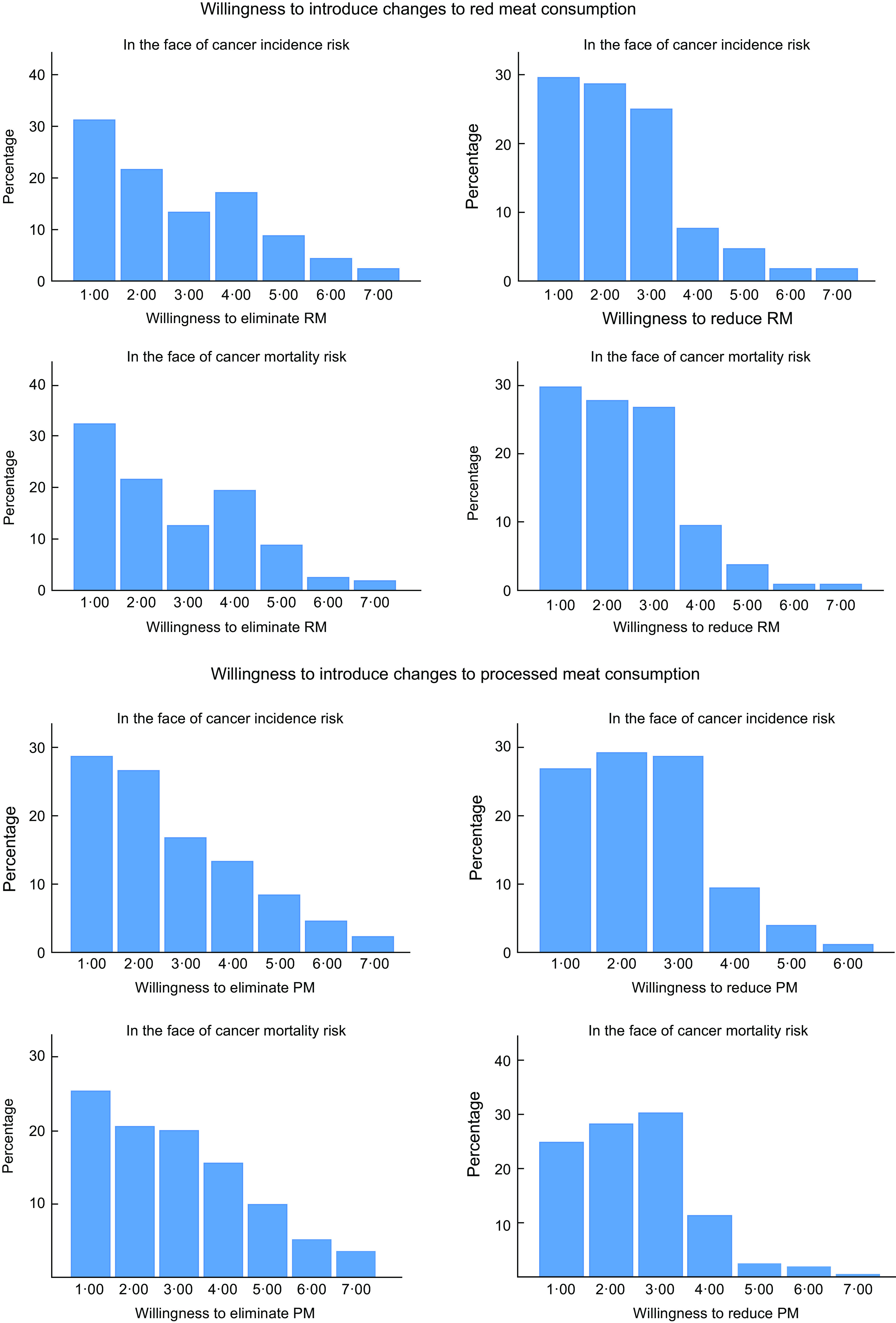
Percentage frequencies of respondents willing and unwilling to introduce any changes in meat consumption in the face of the undesirable cancer health risk

The results concerning the willingness to eliminate or reduce meat consumption were checked for normal distribution using the Kolmogorov–Smirnov test. None of these variables were found to have normal distribution (*P* < 0·001) and are presented in Table 4. The percentage frequency of score occurrences is shown in Fig. 2.

**Table 4:** Willingness to reduce or eliminate red meat or processed meat consumption in the face of undesirable cancer health risk (7-point scale)

Meat consumption	Willingness to change meat consumption in the face of cancer incidence risk			Willingness to change meat consumption in the face of cancer mortality risk		
	n	Median	IQR	n	Median	IQR
Red meat						
Willingness to eliminate	156	2·00	1·00–4·00	157	2·00	1·00–4·00
Willingness to reduce	104	2·00	1·00–3·00	104	2·00	1·00–3·00
Processed meat						
Willingness to eliminate	312	2·00	1·00–4·00	315	3·00	1·00–4·00
Willingness to reduce	222	2·00	1·00–3·00	208	2·00	1·25–3·00

Most respondents were unwilling to eliminate meat consumption in the face of cancer incidence risk for both red (84 %) and processed (84·9 %) meat. Similarly, participants were reluctant to eliminate meat consumption in the face of cancer mortality risk for both red (86·6 %) and processed (81·6 %) meat. The majority of respondents to the question about the willingness to reduce the number of portions of red and processed meat remained unwilling to do so in the face of cancer incidence risk (91·3 % for red meat and 94·6 % for processed meat). In the face of cancer mortality risk, the percentage of negative responses was similar: 94·2 % for red meat and 95·2 % for processed meat.

We performed preliminary analyses using the Pearson chi-squared test to identify associations between individual variables and willingness to change meat consumption habits. The only significant relationships were shown for processed meat (Table 5). The willingness to eliminate in the face of cancer incidence risk was associated with gender and occupational status. Women were more willing to eliminate meat than men (*P* = 0·01). Students showed lower willingness to reduce consumption compared with university employees (*P* = 0·045). Women were also more likely to show willingness to eliminate when faced with the risk of cancer-related death (*P* = 0·006).

**Table 5:** Willingness to eliminate processed meat in the face of cancer health risks according to socio-demographic categories

Parameter		Willingness to eliminate processed meat in the face of cancer incidence risk					Willingness to eliminate processed meat in the face of cancer mortality risk				
		No		Yes		*P* value	No		Yes		*P* value
		*n*	%	*n*	%		*n*	%	*n*	%	
Sex	Male	194	95·1	10	4·9	0·01*	191	93·6	13	6·4	0·006*
	Female	272	88·0	37	12·0		264	85·4	45	14·6	
Age	0–25	190	94·1	12	5·9	0·12	183	90·6	19	9·4	0·54
	26–40	155	89·1	19	10·9		152	87·4	22	12·6	
	≥41	120	88·2	16	11·8		119	87·5	17	12·5	
Education	higher	340	89·9	38	10·1	0·22	331	87·6	47	12·4	0·10
	elementary/secondary	60	96·8	2	3·2		60	96·8	2	3·2	
	post-secondary	66	90·4	7	9·6		64	87·7	9	12·3	
Occupational status	employee	282	88·7	36	11·3	0·045*	277	87·1	41	12·9	0·19
	student	184	94·4	11	5·6		178	91·3	17	8·7	
Religion	Christianity	340	89·2	41	10·8	0·11†	334	87·7	47	12·3	0·48†
	other	9	100·0	0	0·0		9	100·0	0	0·0	
	none	117	95·1	6	4·9		112	91·1	11	8·9	
Family history of oncological diseases	no	241	92·7	19	7·3	0·19	234	90·0	26	10·0	0·42
	yes	225	88·9	28	11·1		221	87·4	32	12·6	

* Significant differences.

† *P* value for the Fisher’s exact test of independence.

A multivariate analysis for willingness to eliminate processed meat in the face of cancer incidence risk was conducted using a logistic regression model with sex, age, education, occupational status, religion and family history of oncological diseases as independent variables. It confirmed the significance of gender; however, occupational status was found to be nonsignificant when these variables were included in the model (Table 6). A similar analysis conducted for willingness to eliminate processed meat in the face of cancer mortality risk also confirmed the significance of gender. Female sex was associated with more than 2-fold higher odds of eliminating meat in the face of cancer incidence risk (*P* = 0·03) and cancer mortality risk (*P* = 0·02), as compared with male sex (Table 6).

**Table 6:** Willingness to eliminate processed meat: logistic regression models

Parameter	Willingness to eliminate processed meat in the face of cancer incidence risk			Willingness to eliminate processed meat in the face of cancer mortality risk		
	OR	95 % CI	*P* value	OR	95 % CI	*P* value
Sex (female)	2·24	1·07, 4·71	0·03*	2·25	1·16, 4·36	0·02*
Age (0–25)	1 (ref.)		–	1(ref.)		–
Age (26–40)	1·21	0·31, 4·68	0·78	0·79	0·26, 2·36	0·67
Age (≥ 41)	1·26	0·31, 5·13	0·75	0·79	0·24, 2·53	0·69
Education (higher)	1(ref.)		–	1 (ref.)		–
Education (elementary/secondary)	0·64	0·12, 3·29	0·59	0·30	0·06, 1·45	0·14
Education (post-secondary)	1·89	0·57, 6·22	0·29	1·15	0·42, 3·13	0·78
Occupational status (student)	0·49	0·12, 2·00	0·32	0·64	0·20, 2·02	0·45
Religion (Christianity)	1(ref.)		–	1(ref.)		–
Religion (other)	NA†		–	NA†		–
Religion (none)	0·47	0·19, 1·17	0·10	0·76	0·38, 1·55	0·46
Family history of oncological diseases (yes)	1·47	0·78, 2·76	0·23	1·20	0·68, 2·12	0·53

* Significant differences.

† Has not been calculated due to 0 cell count (category ‘yes’).

The multivariate logistic model analysis for willingness to reduce processed meat and for willingness to reduce or eliminate red meat revealed no significant associations.

#### Change in meat consumption at 6 months

Among the 176 participants in the follow-up phase, 74 (42 %) declared a change in their eating habits regarding the consumption of red or processed meat since their participation in the main survey. Of the respondents, forty-nine ate less portions of red meat and three consumed more portions of red meat 6 months after the first survey. Similarly, for processed meat consumption, forty-six people declared eating fewer portions, while eleven people ate more portions than reported in the main survey. The most frequent reasons given as the key motivation to change red or processed meat consumption were health (56·8 % and 52·7 %, respectively), environmental issues (24·3 % and 27 %, respectively), animal welfare (24·3 % and 27 %, respectively), preparation time (24·3 % and 18·9 %, respectively) and availability (12·1 % and 18·9 %, respectively). About one-fifth of participants (21·6 %) declared that the change was *definitely* or *possibly* motivated by the information provided during the main survey.

### Qualitative phase

#### Characteristics of participants

Of the 347 individuals who agreed to participate in a face-to-face interview, we included eleven individuals, of whom six were under the age of 35, three were male and seven reported a family history of cancer. The sampled individuals represented various meat consumption (Table 7).

**Table 7:** Characteristics of the study participants of the qualitative interviews

ID	Sex	Age (years)	Education level	Health status/family cancer history	Meat consumption (portions/week)		Willingness to eliminate/reduce red meat	Willingness to eliminate/reduce processed meat	Satisfaction with dietary habits (1–7)
					Red meat	Processed meat			
1	F	29	Higher	?/Y	1–2	3–4	–	4/-	4
2	F	43	Higher	Hashimoto disease/Y	>1	3–4	–	7/-	2
3	M	43	Higher not completed	+/N	3–4	1–2	2/3	–	4
4	F	44	Higher	-/?	>1	5–6	–	2/2	3
5	F	60	Scientific degree	Asthma/Y	3–4	3–4	3/4	6/-	5
6	F	63	Higher	+/Y	3–4	3–4	3/5	5/-	4
7	M	35	Scientific title	+/Y	5–6	7–8	-/-	1/1	4
8	F	32	Higher	+/Y	3–4	3–4	1/1	1/1	5
9	M	20	Higher	+/N	7–8	3–4	2/6	-/-	7
10	F	33	Scientific title	Allergy/Y	1–2	5–6	-/-	4/-	5
11	F	22	Post-secondary	-/?	1–2	>1	-/-	-/-	3

‘+’: no health problems reported; ‘-‘ some health problems reported; ‘Y’- family history of cancer; ‘N’: no family history of cancer; ‘?’: I don’t know.

**Fig. 3:**
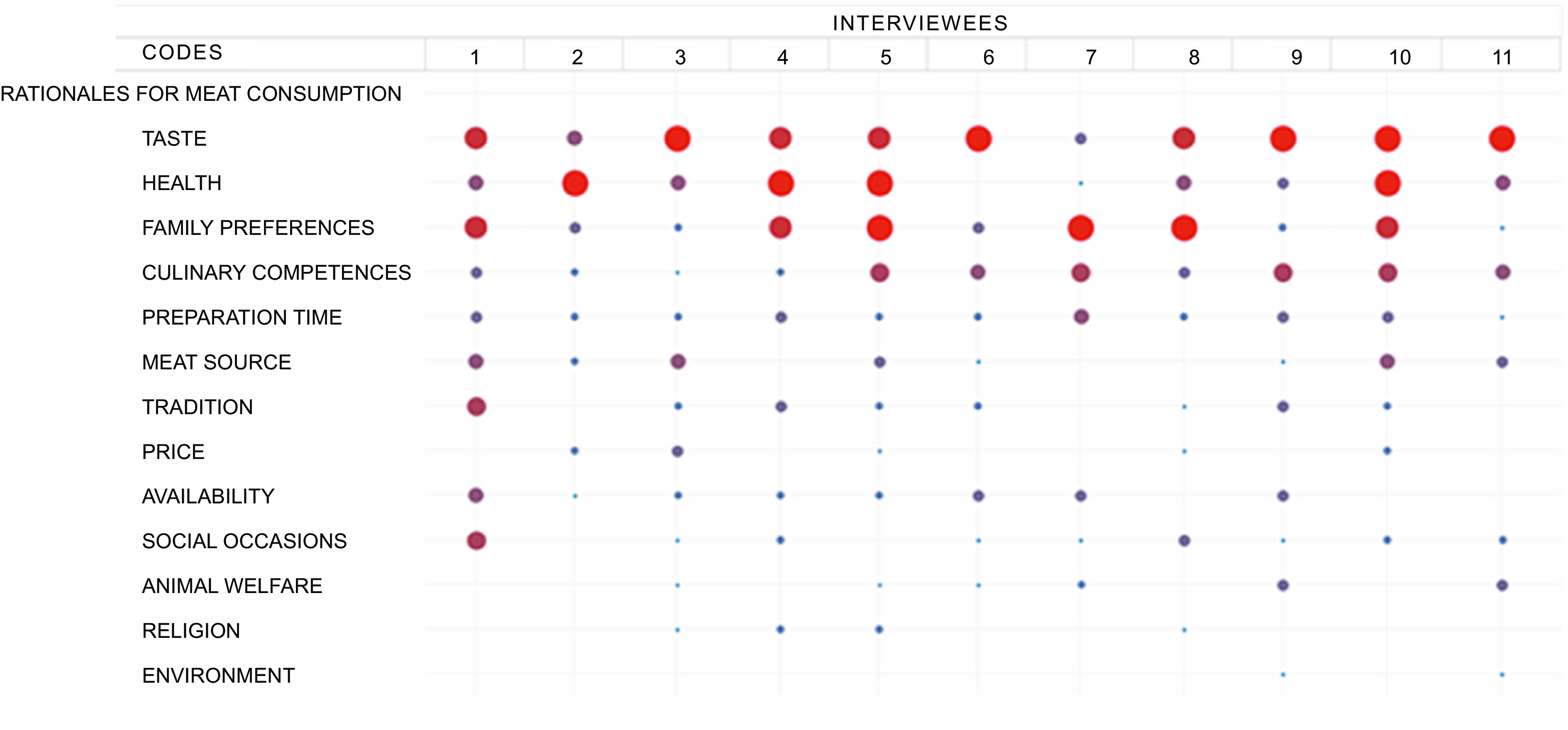
Overview of the rationales for meat consumption discussed during qualitative interviews. Circles represent the coding frequency, and the calculation of the circle size refers to the column. The larger the circle, the more central the rationale was to the interviewee’s individual preferences

The qualitative material reflects a dominant significance of daily dietary habits, well-established eating patterns, as well as health- and food-related convictions and competences. While meat is a common day-to-day and festive food, plant-based food options were considered relatively more demanding to prepare. On the other hand, many interviewees were conscious about the recommendation to reduce meat consumption due to health issues and talked about their efforts to minimise the amount of consumed meat or to buy meat products of good quality. The significance of the various rationales discussed by the interviewees is depicted in Fig. 3.

The subsequent analysis of the relations between the coded segments enabled us to identify four themes reflecting the key motives determining the preferences connected to meat consumption and one overarching theme capturing the relation between them.

#### The importance of taste and texture

When talking about own meat consumption, all of our interviewees referred to the senses, and some of them used very sensual language to describe their attitudes toward meat consumption. A few individuals referred to meat dishes as an object of ‘temptation’ or ‘a culinary delight’ that they could not resist:

*Researcher: What would help you to completely stop [eating meat]? Interviewee: I don’t know if I’d be able to stop. Sometimes I have to, I just have to taste it. (Interviewee_2)*

*I: […] Yes, I feel like I’m addicted to meat [I. and R. laugh]. Therefore, it’s a problem that meat is so appealing to me. Very much so. (Interviewee_4)*

*I am a meat eater … and I’d rather not stop eating meat. Despite my love for animals, I wouldn’t be able to abstain from consuming meat. (Interviewee_6)*

An attachment to meat-based dishes was discussed by most of our interviewees in connection with meat’s unique characteristics, mainly with regard to its taste and texture. Despite current discussions promoting plant-based diets, due to the uniqueness of the taste of meat, most of the interviewees refused to completely eliminate meat from their diet. One of them discussed:

*R: What does meat have that you do not see in vegan or vegetarian dishes? I: Well, that’s a difficult question. I mean, it seems to me that a well-prepared meat dish can do that… Meat easily absorbs the flavours of seasoning. Thus, for example, if you, let’s say, prepare some ribs, and roast them properly, then these herbs and spices are very noticeable at this point. Well, you can, of course, cheat and probably make the same taste somehow different, but something is missing, [maybe it’s] that flavor called ‘umami’, but I guess that’s the point. (Interviewee_9)*

Besides taste, the texture of meat was discussed, a distinguishing mark of dishes containing meat:

*Well, every food has its own specific texture and if we make a dish, it’s nice if each of its elements has its own individual character … and I think it would be difficult to find a substitute for meat that would accurately reflect what the texture of the meat really is like. Well, this is just a characteristic, I would say, that makes eating pleasant when you can differentiate the ingredients of the meal through their own textures. (Interviewee_11)*

These hedonistic motives were presented by some of the interviewees as indisputable reasons that cannot be further negotiated or questioned.

#### Partial health consciousness

As far as the impact of meat consumption on health is concerned, the interviewees talked about two issues. Firstly, they tended to take a stand in the discussion on whether meat is healthy or unhealthy. To support their thesis, most of them mentioned various microelements, for example, proteins, vitamins, micronutrients and purines. The interviewees’ knowledge in this regard, however, seemed incomplete. Their convictions were more consistent regarding meat quality. They talked a lot about the disadvantageous ingredients of meat products, such as additives and preservatives. They were critical about the mass production and the quality of meat products sold in supermarket chains. Most of the interviewees shared their own strategy of coping with the threat of low-quality meat products, for example, reading product etiquette or travelling long distances to buy meat only from known sources, often out of the city and in the countryside. A 44-year-old study participant drives 300 km each month to buy meat from a known source. She said:

*When we return from shopping [in that particular place], half of the freezer gets filled. And there is such a good sausage that my son likes very much. And we buy everything there. Ham with no preservatives, but you have to queue for an hour there as there are so many people shopping there. Well, this is an old proverb—my mother always told me: ‘if you don’t pay a baker, you will have to pay a doctor’. (Interviewee_4)*

When reminded of evidence-based information about the health effects of meat reduction or elimination tailored to the level of meat consumption they had declared in the main survey, the interviewees remained rather sceptical of introducing any dietary changes. Most of them declared that only a more spectacular effect could convince them. One of the interviewees outlined his view:

*R: And what effect would convince you to reduce or eliminate meat consumption? I: Let’s say 50 %. R: So, does that mean that if the results indicated that some 90 fewer people would get cancer that would convince you? I: Exactly. Because then it’s more likely that you could find yourself in one of these groups. Yes. R: And if it weren’t 50 %, just let’s say 40 %, that is, 60-something fewer people would get cancer, would that convince you? I: [pause] It’s hard to say. I suppose that it wouldn’t convince me to stop completely because I already eat meat and it’d be hard to stop. (Interviewee_3)*

For some interviewees, the overall certainty of evidence was more important than the size of the effect itself. The interviewees deliberated that a high or very high certainty of evidence would convince them. For some however, the change would not come without a price:

*R: And if you thought about the certainty of evidence? I: (…) If it was a higher certainty as to the probability of these studies, then maybe I would decide to, even with this effect size. R: We assess the certainty on a 4-point scale. How high on this scale should it be? (…) I: Certain or very certain. (…) R: If the evidence were of a third or fourth degree of certainty, do I understand correctly that it’d be enough to convince you? I: I might take it into consideration. Then I’d be able to eat meat once a day… Once a week at home [laughs] R: And if the effect of reducing or stopping meat consumption was even greater? With a high degree of certainty, would you be ready to stop? D: I would but with a heavy heart. (Interviewee_6)*

#### Habitual nature of meat consumption

A clear pattern blocking the study participants’ willingness to change their omnivorous habits is their cooking competence and socialisation in a particular culinary culture. One of the interviewees, when asked what would help her to reduce your level of consumption of meat, said:

*I: … I think it’d have to be a re-evaluation of my cookbook. What I mean is that, if you’ve been taught since being a child that meat was always there, and there are many recipes with meat and few vegetarian ones … For me, at the moment, trying more vegetarian recipes … is a bit of a pain in the neck. Because I have to try to cook something that, after reading the recipe, I am aware that I might like it—and this is also not obvious—but you know, since childhood you get used to certain flavours … so you won’t always like new dishes that you try. (Interviewee_8)*

A key barrier here seems the conviction that cooking without meat is difficult. Well-known recipes seem quick and simple as opposed to plant-based dishes associated with creativity, sophisticated ingredients and time-consuming culinary procedures. Both female and male interviewees talked about the practical advantages of an omnivorous diet:

*[I like preparing meals with meat], they’re so quick. It only depends on what form I decide to use for cooking. And… well, I often roast or grill meat in the oven. It’s so easy too, and it does the job quickly. (Interviewee_5)*

*Meat is easy to season—you just sprinkle on some pepper and salt and that’s it. However, for plant-based dishes there are various possibilities, and I have the impression that preparing a vegetarian dish requires more creativity and more ingredients are combined. Some kind of pepper, curry, herbs, spices, or whatever …. Those kinds of thing. Meat doesn’t necessarily require this, which, in this respect, makes it easier to prepare a meat dish. It’s fail-safe. (Interviewee_7)*

Another cognitive barrier to going beyond our own culinary habits is the belief that vegetarian meals are not stodgy and would not be accepted by members of the household. Our interviewees often mentioned their closest relatives, when explaining their meat consumption habits. Some specific practices, reoccurring meat dishes, are often attributed to the child’s or spouse’s liking:

*Usually I make stew or spaghetti, which [my son] loves and sometimes stewed pork chops. (…) In our household my son determines what is cooked. If it wasn’t for my son, I wouldn’t eat meat. (Interviewee_2)*

#### Persistence of meat-eating rituals

We observed strong conformism toward including meat dishes in a festive menu. Most of the interviewees took meat as a component of social gatherings for granted.

*For family meetings, let us say, we go to my parents or my in-laws, they prepare a plate, let us say of charcuterie, usually this includes kabanos sausage, ham …. They are cut up into bite-size pieces and you are sitting there, so, you know, you just eat it. (Interviewee_3)*

*Going out, meeting someone or visiting somebody as a guest, we step out of line if we strive to avoid meat dishes. It is hard because it seems to me that it is so ever-present that wherever you socialize with someone, you will find meat. (Interviewee_11)*

For some interviewees, meat during social gatherings is associated with delicacy, being festive and pampering oneself:

*When meeting friends, as one would say, meat rules …. When you enjoy yourself in good company, then culinary pleasure is also important. And it all seems cumulative, right? Because one pleasure is combined with another. Ham rules as a sandwich ingredient and meat for dinner is [obvious]… Few people consciously give up meat. Well, because meat is more expensive, when you are a guest. It was always the meat that was the right choice, at least among my friends. (Interviewee_4)*

Many interviewees took a cold meat platter on a party menu for granted and perceived that being fussy about meat-based dishes when being a guest as socially inappropriate. As hosts, on the other hand, the interviewees tended to willingly follow this pattern or adjust to omnivores’ preferences, for example, due to the preference of male guests to have a fatty snack with alcoholic drinks. Efforts made to introduce vegetarian dishes at barbecues are perceived as a minority preference uniting non-omnivores into a separate category. Restricting the consumption of meat is practised by some interviewees mainly in a religious context. Four interviewees conformed to religious practices of meatless Fridays or avoided meat as part of a resolution for the fasting period of Lent in the 6 weeks running up to Easter.

#### The value conflict: healthy *v*. familiar

Finally, we found that the practices connected to meat preparation and consumption are contextualised by a conflict of two dominant values: what people believe is healthy and what they are familiar with. Valuing well-being and health on the one hand, and being attached to and being bound to practical and sensory preferences rooted in early socialisation, on the other hand, leads to a dissonance. However, this value conflict does not appear to be strong enough to clearly motivate efforts to follow health information and introduce change in meat consumption habits.

## Discussion

When faced with health information about the uncertain reduction in the risk of cancer mortality and incidence, the vast majority of study participants were unwilling to introduce any changes in their consumption habits.

Small differences were observed in regard to the type of meat (red *v*. processed), the health risks reductions (cancer mortality risk *v*. cancer incidence risk) or the extent of the consumption change (stop *v*. reduce). Gender was the significant predictor of the willingness. Women not only tend to be more willing to eliminate and reduce meat when faced with evidence-based information, but also more frequently prioritise practical and less frequently hedonistic reasons to consume meat than men. Moreover, occupational status determined the differences in regard to the willingness to eliminate processed meat in the face of cancer incidence risk. The division between the male and female preferences regarding meat consumption cannot be interpreted without considering the impact that gender norms have on the health-related practices. Our data suggest that women not only consume less portions of meat weekly and significantly more often organise weekly menu than men but they are also more reflective about their dietary habits. Despite being rooted in a particular cultural context, our findings correspond with the studies conducted according to the same main protocol^(31)^. The significance of gender as a key correlate of dietary habits and of the preferences related to meat consumption that we observed is in line with the recent systematic review^(21)^.

Moreover, we observed that meat consumption was closely related to the respondents’ lifestyle. Easy access to meat-based dishes is more important for students than for employees while family preferences determine food choices of people in the older age groups to a greater extent than of those in the younger age group.

Furthermore, we identified four themes reflecting key motives determining meat consumption preferences: the importance of taste and texture, health consciousness, the habitual nature of cooking and persistence of omnivorous habits. The qualitative material reflects also the clash of two conflicting values: health- and food-related convictions and well-established eating habits and sensory and practical preferences. While meat is taken for granted not only as the component of festive menus but also daily food product, plant-based diet appears to be too demanding or not satisfying.

An international comparative study from 2005 revealed that Poles found it more difficult to eat a healthy diet than people in other European countries. While less than a quarter of Dutchmen (20 %), Swedes and Spaniards (21 %), Maltese (23 %), Germans and British people (24 %) indicated that following a healthy diet is difficult, around half of Latvians (48 %), Poles (49 %), Czechs (51 %), Slovaks and Bulgarians (52 %), Hungarians (54 %) and Croatians (57 %) also found it challenging. Among those Poles who found it difficult to eat a healthy diet, 34 % were convinced that preparing a healthy diet takes too much time, 21 % lacked control over what they ate and 20 % lacked information about the food they eat. About 19 % of those who found healthy diet difficult to follow found information about healthy eating contradictory and confusing^(41)^.

Moreover, our interviewees were very conscious about the poor quality of mass-produced meat; nevertheless, they were rather not willing to exclude meat completely from their diet. Instead, they have adopted their shopping strategies to minimise the risk of consuming low-quality products. A similar tendency was observed in a Danish study^(42)^. Furthermore, the participants of our study, similarly to city dwellers surveyed on the determinants of food choices in the central region of Poland^(43)^, did not talk about the environmental consequences of their consumption. The results of the survey by Rejman *et al.*^(43)^ revealed that environmental factors gave way to other determinants such as taste, food quality, healthy ingredients and supporting local food producers. The discourse on the environmental consequences of meat consumption impacted the dietary practices of Poles to a smaller extent than the beliefs and habits of Europeans in other countries. From twenty-five nations surveyed in 2005^(41)^, Poles (68 %) together with Czechs (74 %), Slovaks (73 %) and Estonians (69 %) were the least concerned about the welfare of the animals from which the meat they buy is sourced, in comparison to Swedes (29 %), Greeks (29 %) Luxembourgers (32 %) and Danes (35 %), who most often reported paying attention to the welfare of farmed animals^(41)^. Ten years later, attitudes toward animal welfare in Poland were still less clear than in other countries. Every eighth Pole was not able to relate to any of the four statements describing animal welfare. While almost every respondent in Sweden (99 %), Finland (99 %) and Portugal (99 %) considered animal welfare to be ‘important’, Poles (86 %), Hungarians (86 %) and Croatians (86 %) were the most sceptical about it^(44)^.

Similar to the findings from a systematic review^(21)^ as well as from another study^(45)^, the majority of our participants were reluctant to change their meat consumption habits. The evidence-based information about the health effect of reducing or eliminating meat consumption was not convincing enough to make any changes. Even the interviewees who appreciated the information were not interested in changing their habits.

### Strengths and limitations

Our study addressed the overlooked dimension of developing nutritional recommendations, and its findings can inform nutritional guidelines regarding meat consumption. However, the collected material was limited to presenting health benefits related to two cancer-related outcomes and omitted the possible concomitant benefits on cardiovascular risk reduction.

Moreover, the mixed methods approach applied here can be used in analogous inquiries aiming at gaining understanding of culturally determined values and preferences toward other key products. Moreover, further studies are needed to determine the preferences of individuals without the academic background and those consuming less than three servings of meat per week.

## Conclusions

University students and employees are unwilling to introduce changes even when faced with evidence-based health information about the uncertain reduction in the risk of cancer mortality and incidence. Women are statistically more willing to eliminate processed meat when faced with evidence-based information than men. Students appeared to be less willing to eliminate processed meat consumption compared with university employees, but after standardising on other variables in the logistic regression model, the relationship was no longer significant. Furthermore, dietary habits are rooted in gender norms, scenarios of social gatherings and lifestyles. Meat consumption is entangled in the value conflict, in which health consciousness is challenged by the importance of meat taste and texture, the habitual nature of cooking and persistence of omnivorous habits.

Our study has significant implications for future development of dietary recommendations that add to the ongoing discussions on how to improve the implementation of dietary guidelines^(46)^. Our findings emphasise the importance of accounting for cultural determinants and individual preferences shaping dietary patterns. Guideline panels should consider that values and preferences linked to meat consumption are rather stable and evidence-based health information appears to many to be insufficiently persuasive to encourage a dietary change.

## Supplementary material

For supplementary material accompanying this paper visit https://doi.org/10.1017/S1368980022000866. click here to view supplementary material
