# Supplementary material for: Values and preferences influencing willingness to change red and processed meat consumption in response to evidence-based information: a mixed methods study
Source: Public Health Nutr. 2022 Apr 8;25(8):2084–98. doi: 10.1017/S1368980022000866 (PMC9991620; doi:10.1017/S1368980022000866)
Supplement: Supplementary file 1 [file S1368980022000866sup.zip › S1368980022000866sup002.doc]

Supplementary material 2
