# Supplementary material for: Values and preferences influencing willingness to change red and processed meat consumption in response to evidence-based information: a mixed methods study
Source: Public Health Nutr. 2022 Apr 8;25(8):2084–98. doi: 10.1017/S1368980022000866 (PMC9991620; doi:10.1017/S1368980022000866)
Supplement: Supplementary file 1 [file S1368980022000866sup.zip › S1368980022000866sup003.doc]

## **Supplementary material** 4

### Introduction

My name is _________________. I work in _________________. We are meeting regarding the study that you participated in on _________________ [date]. You have completed the electronic questionnaire on meat consumption.

You have provided your consent to participate in a face-to-face interview, in which I would like you to tell me more about your food preferences. This conversation will take about 45 to 60 minutes. You can choose to quit at any time without giving a reason or to refuse to answer a given question. For the purpose of the analysis, it is important for me to be able to record our conversation. The collected data are confidential and will only be used for scientific purposes. If you confirm your consent to participate in the study, please sign the consent form.

Do you remember this survey?

*If yes:* Reflecting on that study, do you have any thoughts on the study itself or how it influenced your nutrition? *Show the pictures tailored to the interviewee’s responses from the main survey*.

*If not:* Remind the interviewee what the survey was about and what responses he or she provided.

Let me remind you that the study concerned the consumption of two types of meat: unprocessed red and processed meat. By unprocessed red meat, we mean the following types of meat: beef, veal, pork, lamb, lamb, horse, and goat, with the exclusion of poultry, fish, and seafood. When we ask about the number of servings of unprocessed red meat, we mean a 120-gram serving that is similar in size to a deck of cards. Red processed meat means meat preserved by smoking, curing, or salting or by adding preservatives such as sausages, bacon, and cold cuts. When we ask about the number of servings of processed meat, we mean a serving of 50 to 75 grams, which is similar in size to about half a deck of cards.

### Part A

A. UNPROCESSED MEAT

In the survey, you declared that you eat meat. I would like to know what are your main reasons for consuming red unprocessed meat?

*If the respondent does not mention the reasons reported in the survey, continue as follows:*

*In the questionnaire, you also mentioned other issues (relevant data from the database)*

*x_________________*

*y_________________*

*z_________________*

How does X/Y/Z affect your consumption of unprocessed red meat?

*Probes:*

For what reasons do you decide to eat unprocessed red meat?

Under what circumstances do you eat dishes with red meat?

B. PROCESSED MEAT [AV]

In the survey, you declared that you eat processed meat. I would like to know what are your main reasons for eating red processed meat?

*If the respondent does not mention the reasons reported in the survey, continue as follows:*

*In the questionnaire, you also mentioned other issues (relevant data from the database)*

*x_________________*

*y_________________*

*with_________________*

How does X/Y/Z affect your consumption of processed meat?

*Probes:*

For what reasons do you decide to eat processed meat?

Under what circumstances do you eat dishes with processed meat?

### Part B

Three months ago, you declared that you ate _____ servings of unprocessed red meat [*relevant data from the database*] and/or ______ servings of processed meat [*relevant data from the database*] per week on average.

Has anything changed in this regard?

*If yes:* what has changed and why? Was it the health information you received during the study three months ago that changed your meat consumption?

*If not:* In the survey, we asked you about your readiness to reduce the amount of meat portions after reviewing the survey results. How did the survey influence the way you think about meat consumption?

What could help you reduce or eliminate meat consumption?

*Probes:*

What would make it difficult for you to reduce or eliminate meat consumption?

What made you not believe the presented data?
