# Supplementary material for: Values and preferences influencing willingness to change red and processed meat consumption in response to evidence-based information: a mixed methods study
Source: Public Health Nutr. 2022 Apr 8;25(8):2084–98. doi: 10.1017/S1368980022000866 (PMC9991620; doi:10.1017/S1368980022000866)
Supplement: Supplementary file 1 [file S1368980022000866sup.zip › S1368980022000866sup004.doc]

## **Supplementary material** 3

Dear Sir/Madame,

Although there is a substantial body of knowledge about the impact of food on health, it can sometimes seem ambiguous. Participation in this study gives you an opportunity to learn about the latest research findings on the effect of meat consumption on health.

We are an interdisciplinary team of researchers led by dr hab. Małgorzata Bała, a professor at Jagiellonian University, who are interested in learning about individual preferences with regard to food choices. As part of our research project, we collaborate with universities in Spain, Canada, and Brazil in a common effort to identify factors that determine individual dietary decisions, especially those concerning meat consumption.

The survey consists of three parts. The first part is designed to provide basic information about you as a participant, the second part contains questions about your preferences as to nutrition, and the third part presents research conclusions regarding the impact of meat consumption on health and contains questions asking about your attitude toward these conclusions.

In the study, you will be asked about the consumption of unprocessed red and processed meat. By unprocessed red meat, we mean the following types of meat: beef, veal, pork, lamb, horse, and goat. Unprocessed poultry, fish, and seafood were excluded. For the purpose of this study, a serving of red unprocessed meat was defined as a 120-gram serving that is similar in size to a deck of cards. By processed meat, we mean meat preserved by smoking, curing, or salting or by adding preservatives such as sausages, bacon, or cold cuts. A serving of processed meat was defined as a 50-gram serving that is similar in size to about half of a deck of cards.

### Introductory questions

A1. Please indicate whether you belong to any of the following groups of people requiring special dietary recommendations:

- people with a history of cancer

- people with a history of severe cardiovascular events (stroke, myocardial infarction, heart failure, symptomatic peripheral arterial disease)

- people who changed meat consumption habits or lifestyle (e.g., intense sports) due to other diseases or health conditions (eg, anemia, kidney disease, digestive disorders)

- pregnant women

- vegetarians (a vegetarian diet excludes red meat, chicken, and fish; some vegetarians choose to eat eggs and dairy, while others also exclude these products).

- vegans (a vegan diet includes grains, vegetables, legumes, and nuts and excludes meat, fish, poultry, dairy products, eggs, and any products containing these ingredients)

- people under the age of 18 years

- people over the age of 80 years

| Yes | No |
| --- | --- |

A2. Do you usually eat at least 3 servings of unprocessed red meat or processed meat per week?

| Yes | No |
| --- | --- |

### Informed consent form

I hereby consent to the participation in the study "Willingness to change consumption habits among employees and students at Krakow universities" conducted under the supervision of dr hab. Małgorzata Bała, Professor at the Department of Hygiene and Dietetics of Jagiellonian University Medical College. I declare that I have been informed about the purpose of the study, voluntary participation, the possibility to ask questions and obtain answers from the person providing information about the study, the ways the collected material will be used, and the possibility to quit the study at any stage.

| Yes | No |
| --- | --- |

### Consent to the processing of personal data

I hereby consent to the processing of my personal data **for the sole purposes of the study** on the willingness to change **meat** consumption habits and to the use of study results in scientific work, in accordance with the Regulation of the European Parliament and Council (EU) 2016/679 of 27 April 2016 and in accordance with the information clause attached to my consent.

| Yes | No |
| --- | --- |

### Part One: Personal data

| 1. Age |  |
| --- | --- |
| 2. Sex | ☐Male  ☐Female |
| 3. Weight (kg) |  |
| 4. Height (cm) |  |
| 5. Ethnicity | ☐ Polish  ☐ Belarusian  ☐ Lithuanian  ☐ Romani  ☐ Ukrainian  ☐ Lemko  ☐ Silesia  ☐ Russian  ☐ German  ☐ Jewish  ☐ French  ☐ English  ☐ Italian  ☐ Kashubian  ☐ American  ☐ Other, please specify:__________________ |
| 6. Level of education | ☐ less than primary  ☐ primary education  ☐ secondary education  ☐ Bachelor’s degree  ☐ Master’s degree  ☐ Doctorate degree and higher |
| 7. Employment status | ☐ Employed for wages  ☐ Self-employed  ☐ Out of work and looking for work  ☐ Out of work but not currently looking for work  ☐ Homemaker  ☐ Student  ☐ Military  ☐ Retired  ☐ Unable to work |
| 8. Marital status | ☐ Single  ☐ Married  ☐ Living common law  ☐ Separated  ☐ Divorced  ☐ Widowed |
| 9. Family characteristics | ☐ No children  ☐ 1 child  ☐ 2 children  ☐ 3 or more children |
| 10. How often do you exercise or play sport? | ☐ every day  ☐ at least once a week  ☐ at least once a month  ☐ occasionally (for example skiing)  ☐ never  ☐ don’t know |
| 11. How often do you do other physical activities such as cycling from one place to another; gardening; dancing, etc.? | ☐ every day  ☐ at least once a week  ☐ at least once a month  ☐ occasionally  ☐ never  ☐ don’t know |
| 12. Religious beliefs | ☐ Christian  ☐ Islamic  ☐ Judaism  ☐ Hindus  ☐ Buddhist  ☐ None  ☐ Other, please specify: ______________ |
| 13. Chronic health conditions | ☐ none  ☐ musculoskeletal disorders  ☐ nervous system disorders  ☐ renal and urological disorders  ☐ digestive tract disorders  ☐ hormone system disorders  ☐ Other, please specify:___________________ |
| 14. Family history of cancer | ☐ don’t know ☐ none ☐ yes, please fill out the table below where it applies: \| Breast cancer \| Colorectal Cancer \| Ovarian cancer \| Other specify; Parents \| \| \| \|; Grandparents \| \| \| \|; Siblings \| \| \| \|; Aunts/Uncles \| \| \| \|; Cousins \| \| \| \|; Other, specify \| \| \| \| |

### Part Two: Meat consumption habits

In this part of the study, we will ask about your dietary preferences.

*Pictures illustrating PM and RM and portion sizes (Appendix 1)*

**1.** How many servings of **unprocessed red meat** and **processed meat** do you usually eat in a week?

| **TYPES OF MEAT** | **Average consumption of servings in a week** | | | | | | | | |
| --- | --- | --- | --- | --- | --- | --- | --- | --- | --- |
|  | **<1** | **1-2** | **3-4** | **5-6** | **7-8** | **9-10** | **11-12** | **13-14** | **>15** |
| **Unprocessed red meat**  ***Eg. Beef filet, pork chop***  (a serving size is equal to 120 grams) |  |  |  |  |  |  |  |  |  |
| **Processed meat**  ***Eg. Sausages, bacon.*** (a serving size is equal to 50 grams). |  |  |  |  |  |  |  |  |  |

**2.** What types of **unprocessed red meat** do you usually eat most? Please select up to 3 response options.

1. steak
2. cutlet
3. goulash
4. roast
5. meatball
6. others, please specify

**3.** What types of **processed meat** do you usually eat most? Please select up to 3 response options.

1. sausage
2. ham and cold cuts
3. salami
4. pate
5. kabanos
6. bacon
7. preserved or canned meat
8. other, please specify

**4.** Of the factors listed in the first column, which are the most important in determining your usual consumption of **unprocessed red meat** and **processed meat**? (Select up to 3 per type of meat)

|  | **Unprocessed red meat**  ***Eg. beef, pork, veal, lamb products, e.g. steak, cutlet, stew, roast, pulp***  (a serving size is equal to 120 grams). | **Processed meat**  ***Eg. e.g. sausage, sausage, ham and cold cuts, salami, pate, kabanos, bacon, meat or canned food***  (a serving size is equal to 50 grams). |
| --- | --- | --- |
| **Cost**  *I can afford to buy it* |  |  |
| **Taste**  *I like the taste of it* |  |  |
| **Availability**  *I can find the selection of the products I want* |  |  |
| **Family preference**  *My family favours its consumption* |  |  |
| **Tradition**  *It's part of my tradition* |  |  |
| **Religion**  *My religion favours its consumption* |  |  |
| **Cooking time**  *I have the time to cook it* |  |  |
| **Social context**  *I consume it in social contexts like barbecues* |  |  |
| **Animal welfare issues**  *I consume animal welfare products. For example, they have been fed on grassland, bred outdoors and have been well-treated at the slaughterhouse, transported and priced.* |  |  |
| **Environmental issues**  *I consume products with a low environmental impact to favor the environment.* |  |  |
| **Other, please specify:** |  |  |

**5.** Where do you get your nutritional information? Select all that apply

a. Internet articles (i.e. culinary blogs)
b. Friends, family, or neighbours
c. Physicians or other healthcare professionals
d. Publications by Instytutu Żywności i Żywieni
e. Other sources (please specify)____________________________________
f. None, I don’t get any nutritional information of what I eat

**6.** Who in your household is usually responsible for the activities related to the process of preparing the meals? Please mark who is responsible for each of the four activities listed in the first column.

| **ACTIVITIES** | **RESPONSABLE/S** | | | | |
| --- | --- | --- | --- | --- | --- |
|  | Most of the time I do it on my own | Most of the time I share this responsibility with somebody else (e.g. spouse) | Most of the time somebody else from my household does it (e.g. spouse) | Most of the time by a third party (catering service, social workers, etc.) | Most of the time I eat out |
| Shopping for food |  |  |  |  |  |
| Deciding the daily menu |  |  |  |  |  |
| Cooking during the week days |  |  |  |  |  |
| Cooking on special occasions (weekends, parties, family celebrations) |  |  |  |  |  |

**7.** How often do you eat alone?

a. Always
b. Most of the times
c. Sometimes
d. Rarely
e. Never

**8.** Do you read labels when purchasing unprocessed red meat and processed meat? Select the most important option reflecting your experience per type of meat (If your answer is NO, skip the next question and go to question nº 10)

|  | **Unprocessed red meat**  ***Eg. Beef filet, pork chop***  (a serving size is equal to 120 grams). | **Processed meat**  ***Eg. Sausages, bacon.*** (a serving size is equal to 50 grams). |
| --- | --- | --- |
| a) Yes, always |  |  |
| b) Yes, most of the times |  |  |
| c) Yes, sometimes |  |  |
| d) Yes, rarely |  |  |
| e) No, never |  |  |
| f) No, labels are not shown |  |  |

**9.** If yes, what do you usually look for on labels? If you answered “No, never”, skip to question nº10

| **Information on labels** | **Unprocessed red meat**  ***Eg. Beef filet, pork chop***  (a serving size is equal to 120 grams). | **Processed meat**  ***Eg. Sausages, bacon.*** (a serving size is equal to 50 grams). |
| --- | --- | --- |
| a. Price |  |  |
| b. Nutritional value |  |  |
| c. Ingreditens |  |  |
| d. Origin |  |  |
| e. Expiration date |  |  |
| f. Other, please specify |  |  |

**10.** Have you ever considered about making changes about your eating habits recently? Select the most important option reflecting your experience

a. Yes, once
b. Yes, a few times
c. Yes, I do it constantly
d. No, never

**11.** If yes, what kind of changes have you been considering to make? Select all that apply

a) Reducing amount of processed foods, i.e. cookies
b) Reducing amount of saturated fats, i.e. sausages, cheese
c) Reducing amount of carbohydrate, i.e. white bread
d) Reducing amount of sugar-sweetened beverages, i.e. soda drinks (e.g. Sprite etc.)
e) Increasing amount of super foods, i.e. chia, kale
f) Increasing amount of fruits and vegetables
g) Increasing amount of whole wheat products, seeds and grains

Others, if so, please specify:________________________________________________

**12.** On a scale from 1 (definitely dissatisfied) to 7 (definitely satisfied), please indicate to what extent are you satisfied with your current eating habits?

**13.** Over the last year, have you thought about changing your eating habits?

1. Yes, I think about it all the time

2. So many times

3. Yes, occasionally

4. No, never

**14.** If so, what did you want to change? Please circle all the answers that reflect your experience.

1. Reduce the amount of certain products, e.g. sweets
2. Reduce the amount of saturated fat, e.g. sausages, cheese
3. Reduce the amount of carbohydrates, e.g. white bread
4. Reduce the amount of sweetened drinks, e.g. orangeade, Sprite
5. super-food, e.g. chia seeds, kale
6. Increase the amount of fruit and vegetables
7. Increase the amount of whole grains, seeds and grains
8. Other, please specify ________________________________________________

### Part Three

One way to study the health effects of eating a variety of foods is to determine the prevalence of disease or death from it.

|  | **Cancer mortality** | | | |
| --- | --- | --- | --- | --- |
|  | **Symptoms & Signs** | | - Before you die you experience symptoms related to cancer and its spread, such as pain, weakness/fatigue; those symptoms may have various duration, you may suffer those symptoms for several years - Before you die you experience unwanted effects of treatment you received for cancer. - You are dead and you do not feel any pain or breathlessness. | |
|  | **Treatment** | | - There is no need for any treatments and they are stopped | |
|  | **Consequences** | | - You lose your vital bodily and mental functions, ending your life. - You will leave everything that was important in short time span. | |
| **Cancer incidence** | | | |  |
| **Symptoms & Signs** | | - Cancer is wide group of diseases and may cause many signs or symptoms - Some signs and symptoms are common for different cancers while other more specific for each type of cancer - Not explained loss in body weight, night sweats, fever - Problems with eating, loss of appetite - Weakness/ fatigue - Sometimes bleeding or discharge, blood in stool or urine - Change in bowel habits, difficult or painful urination - Pain - Unexplained anaemia - Persistent cough or blood in saliva - Persistent lumps or swollen glands - Changes on the skin | |  |
| **Treatment** | | - There are different types of treatment that will depend on the type of cancer and how the cancer is advanced. - You may receive only one treatment, but in most cases a combination of subsequent is needed: surgery and/or hormone therapy (giving hormones or drugs that block hormones to slow down cancer growth), chemo or immunotherapy (drugs that kill cancer cells or flag them for immune system to destroy) and/or radiation therapy (radiation in high doses to kill cancer cells or slow their growth). | |  |
| **Consequences** | | - You can experience side effects of cancer treatment, such as anaemia, loss of appetite, fatigue, hair loss, nausea - You can experience pain, gastrointestinal problems, urinary problems - It will affect your social life short term and possibly long term - You can experience long-term consequences of cancer and its treatment, such as problems with heart, lungs, endocrine system, bones and joints, digestion, memory - You may experience anxiety, depression and other emotional problems - You may no longer be able to participate in your regular activities - You may die | |  |

*A video presenting explanation presented also in written below.*

Based on our study, for people like you consuming 3 servings per week of processed meat, the risk of developing cancer is 18.5% and the risk of dying from cancer is 10.5%. This means that out of 1,000 people approximately 185 will develop cancer and 815 will not and 105 will die from cancer and 895 will not.

For people consuming 3 fewer weekly servings of processed meat, the risk of developing cancer is 18.3% and the risk of dying from cancer is 9.7%. This means that out of 1,000 people approximately 183 will develop cancer and 817 will not, and 96 will die from cancer and 903 will not. Overall, 2 fewer people will develop cancer and 8 fewer people will die from cancer when consuming 3 fewer weekly servings of processed meat compared to not decreasing their processed meat intake”.

In other words, a reduction of 3 servings per week of processed meat may reduce the risk of developing or dying from cancer and the certainty around this effect is very low to low. This means that the results that we have shown you come from studies with important limitations. Because of this, it remains possible that reducing your meat consumption will have no benefits in reducing cancer risk or dying from cancer.

*Tailored set of Magics (Supplementary materials 2)*

Given what is presented in the picture, on a scale from **1 (meaning definitely not**) to **7 (meaning definitely yes),** would this **uncertain** reduction in the **risk of developing cancer** and the **risk of dying from cancer** be enough for you to stop eating processed meat?

| **1** | **2** | **3** | **4** | **5** | **6** | **7** |
| --- | --- | --- | --- | --- | --- | --- |
| Definitely not,  this information is not convincing me at all to stop eating processed meat |  |  |  |  |  | Definitely yes**,** this information is totally convincing me to stop eating processed meat |
| ☐ | ☐ | ☐ | ☐ | ☐ | ☐ | ☐ |

*Only to those respondents unwilling to stop eating meat (≤4 from the Likert-scale):*

On a scale from **1(meaning definitely not)** to **7 (meaning definitely yes)**, would this **uncertain** reduction in the **risk of developing cancer** and the **risk of dying from cancer** be enough for you to reduce your processed meat intake? Please, consider that the **possible** cancer risk reduction you might achieve will be smaller by reducing your meat intake than stopping it completely

| **1** | **2** | **3** | **4** | **5** | **6** | **7** |
| --- | --- | --- | --- | --- | --- | --- |
| Definitely not,  this information is not convincing me at all to reduce eating processed meat |  |  |  |  |  | Definitely yes**,** this information is totally convincing me to reduce eating processed meat |
| ☐ | ☐ | ☐ | ☐ | ☐ | ☐ | ☐ |

**Consent for further contact**

Do you agree to be contacted again in 3 months in relation to the second part of the survey, including a few short questions in the form of an in the online survey? **Yes No**

Do you consent to participate in the qualitative survey in the form of an interview with the researcher, which will be carried out in the coming weeks? **Yes No**

Please enter your preferred method of contact (e-mail, phone) and convenient time to be contacted:

Phone number	__________________________________

E-mail address	___________________________________

Convenient time to be contacted___________________________________
